# Insights into the Oncogenic, Prognostic, and Immunological Role of BRIP1 in Pan-Cancer: A Comprehensive Data-Mining-Based Study

**DOI:** 10.1155/2023/4104639

**Published:** 2023-04-28

**Authors:** Yongru Liu, Xi Wu, Yunlu Feng, Qingwei Jiang, Shengyu Zhang, Qiang Wang, Aiming Yang

**Affiliations:** Department of Gastroenterology, State Key Laboratory of Complex Severe and Rare Diseases, Peking Union Medical College Hospital, Peking Union Medical College and Chinese Academy of Medical Sciences, Beijing 100730, China

## Abstract

**Background:**

BRCA1 interacting helicase 1 (BRIP1), an ATP-dependent DNA helicase which belongs to an Iron-Sulfur (Fe-S) helicase cluster family with a DEAH domain, plays a key role in DNA damage and repair, Fanconi anemia, and development of several cancers including breast and ovarian cancer. However, its role in pan-cancer remains largely unknown.

**Methods:**

BRIP1 expression data of tumor and normal tissues were downloaded from the Cancer Genome Atlas, Genotype-Tissue Expression, and Human Protein Atlas databases. Correlation between BRIP1 and prognosis, genomic alterations, and copy number variation (CNV) as well as methylation in pan-cancer were further analyzed. Protein-protein interaction (PPI) and gene set enrichment and variation analysis (GSEA and GSVA) were performed to identify the potential pathways and functions of BRIP1. Besides, BRIP1 correlations with tumor microenvironment (TME), immune infiltration, immune-related genes, tumor mutation burden (TMB), microsatellite instability (MSI), and immunotherapy as well as antitumor drugs were explored in pan-cancer.

**Results:**

Differential analyses showed an increased expression of BRIP1 in 28 cancer types and its aberrant expression could be an indicator for prognosis in most cancers. Among the various mutation types of BRIP1 in pan-cancer, amplification was the most common type. BRIP1 expression had a significant correlation with CNV and DNA methylation in 23 tumor types and 16 tumor types, respectively. PPI, GSEA, and GSVA results validated the association between BRIP1 and DNA damage and repair, cell cycle, and metabolism. In addition, the expression of BRIP1 and its correlation with TME, immune-infiltrating cells, immune-related genes, TMB, and MSI as well as a variety of antitumor drugs and immunotherapy were confirmed.

**Conclusions:**

Our study indicates that BRIP1 plays an imperative role in the tumorigenesis and immunity of various tumors. It may not only serve as a diagnostic and prognostic biomarker but also can be a predictor for drug sensitivity and immunoreaction during antitumor treatment in pan-cancer.

## 1. Introduction

Cancer remains a thorny problem which brings immense suffering to individual health and financial burden. Despite the tremendous advances in the detection of novel biomarkers and development of targeted drugs as well as immunotherapies in recent decades, the high morbidity and mortality of cancer is still frustrating. According to the GLOBOCAN 2020 statistics, there were approximately 19.3 million new cases and 10.0 million deaths related to cancer worldwide in 2020, and the global cancer burden was expected to reach 28.4 million cases in 2040 with a rise of 47% from 2020 [1]. Therefore, persistent efforts are urgently needed to understand the complex mechanisms of tumorigenesis and identify novel biomarkers for early diagnosis, clinical prognosis, and therapy response. Thanks to various public databases, valuable data can be mined and pan-cancer analysis can be conducted for a comprehensive investigation of extracted genes.

BRIP1 (BRCA1 interacting helicase 1), also known as FANCJ (as the gene mutated in the J complementation group of Fanconi anemia) or BACH1 (BRCA1-associated C-terminal helicase), was first discovered in 2001 by its interaction with BRCA1 [2]. BRIP1 is a protein coding gene which encodes for homologous recombination repair (HRR)-related protein and facilitates DNA single-strand break (SSB) and DNA double-strand break (DSB) repair during vital biological processes including DNA replication, transcriptional regulation, and overall metabolic health [3]. BRIP1, whose encoded protein belongs to an Iron-Sulfur (Fe-S) helicase cluster family with a DEAH domain, helps to preserve chromatin structure and function and may also maintain genomic and epigenetic stability. Besides its collaboration with numerous DNA metabolizing proteins implicated in the detection and repair of DNA damage, BRIP1 also participates in cell cycle checkpoint control [4]. Recent studies manifest that BRIP1 took part in miscellaneous tumorigeneses and pathological conditions. The National Comprehensive Cancer Network (NCCN) guidelines identified BRIP1 as a potential risk factor for breast cancer, especially for triple negative breast cancers [5]. In ovarian cancer, a deleterious mutation of BRIP1 was associated with low-grade histology and led to an increased risk of the disease [6]. In endometrial cancer, BRIP1 correlated to tumor recurrence and patients with mutations in BRIP1 might benefit from poly ADP-ribose polymerase (PARP) inhibitors [7]. Mikaeel et al. reported that BRIP1 might be a cancer-predisposing gene in young-onset colorectal cancer [8]. Mani et al. suggested that BRIP1 was of the imperative role in maintaining neuronal cell health and homeostasis by suppressing oxidative stress, excitotoxicity induced DNA damage, and protecting mitochondrial integrity [3]. However, there is a lack of a comprehensive pan-cancer analysis of BRIP1. Hence, we extracted diverse data from The Cancer Genome Atlas (TCGA), Genotype-Tissue Expression (GTEx), Cancer Cell Line Encyclopedia (CCLE), Human Protein Atlas (HPA), cBioPortal and GeneMANIA databases and evaluated the expression, prognosis, and mutation as well as function of BRIP1 in various cancer types. We further carried out immune infiltration analysis, and the relationships between BRIP1 and immune-related genes and tumor mutation burden (TMB)-microsatellite instability (MSI) as well as immunotherapy and targeted drug responses were subsequently analyzed. This in-depth data-mining based study helped us understand the role of BRIP1 in tumorigenesis, provided evidence for its diagnostic and prognostic evaluation in the clinic, and shed light on the novel targeted treatment as well as immunotherapy in pan-cancer.

## 2. Materials and Methods

### 2.1. Raw Data Collection and Differential Expression Analysis

The mRNA expression profiles and related clinical information of 33 human cancers and their corresponding normal samples were, respectively, downloaded from TCGA via the UCSC Xena platform (https://xena.ucsc.edu/) [9]. Additional gene expression data were also retrieved from GTEx (https://gtexportal.org/home/datasets) and CCLE (https://sites.broadinstitute.org/ccle). BRIP1 expression was transferred to transcripts per million (TPM) and then evaluated by log_2_ transformation. *T*-test was carried out to identify its different expression between tumor and normal tissues as well as between different TNM stages. R software (Version 4.0.3, https://www.Rproject.org) and the “ggplot2” R package (Version 3.3.3) were applied to analyze the data and draw box diagrams. The abbreviations and full names of the various cancer types were listed in Table 1. Besides, to evaluate the differential expression of BRIP1 at the protein level, immunohistochemistry (IHC) images in multiple tumors and normal tissues were downloaded from HPA (https://www.proteinatlas.org/). The antibody used for IHC was HPA005474.

### 2.2. Prognostic Value of BRIP1 in Pan-Cancer

Overall survival (OS), disease-specific survival (DSS), disease-free interval (DFI), and progression-free interval (PFI) were of vital importance in exploring the association between BRIP1 expression and prognosis. Related survival data were downloaded from the UCSC Xena platform. The Kaplan–Meier (KM) method and log-rank test were utilized to carry out survival analyses in each cancer with the best cut-off value of BRIP1 expression by using R packages “survminer” and “survival.” Univariate Cox regression and R package “forestplot” were also used to identify the relevancy between BRIP1 expression and survival in pan-cancer. The hazard ratio (HR) and Cox's regression *P* values were shown in the plot.

### 2.3. BRIP1 Mutation and Its Correlation with Copy Number Variation and DNA Methylation

To further investigate the modification of BRIP1 gene in pan-cancer, we used the cBioPortal database (https://cbioportal.org) to explore its mutation, structural variant, amplification, deep deletion, and multiple alterations [10]. As copy number variation (CNV) and copy number alteration (CNA) played a critical role in cancer initiation and progression, and promoter methylation was critical in gene silencing and inactivation, related data were downloaded from cBioPortal for further analyses. Association between the expression of BRIP1 and CNV as well as promoter methylation was further evaluated by carrying out Pearson correlation analysis. R software and the “ggplot2” R package were acquired to analyze the data and draw lollipop plots.

### 2.4. Gene Interaction of BRIP1 and Its Enrichment and Variation Analysis

The GeneMANIA database (https://www.genemania.org) was applied to detect functionally similar genes to BRIP1 and construct the protein-protein interaction (PPI) network [11, 12]. Subsequently, gene set enrichment analysis (GSEA) was performed in pan-cancer based on the Gene Ontology (GO) and Kyoto Encyclopedia of Genes and Genomes (KEGG) database to explore the biological signalling pathway by using R package “clusterProfiler,” and R package “ridgeplot” was used to draw the ridge plot [13, 14]. We further downloaded the “gmt” file of the 50 hallmark gene sets from the Molecular Signatures Database (MSigDB, via https://www.gsea-msigdb.org/gsea/msigdb/index.jsp) [15, 16] and performed gene set variation analysis (GSVA) using the “GSVA” R package to explore the correlation between BRIP1 expression and 50 hallmark pathways. Pearson correlation analysis was conducted, and the “pheatmap” R package was used to turn the results into heatmap.

### 2.5. BRIP1 Expression and Its Relationship with Immunity

Tumor microenvironment (TME), a crucial element of tumor, has been reported to play a decisive role in cancer development and therapeutic responses. Hence, we carried out evaluation of the association between BRIP1 expression and the proportion of immune-stromal component in pan-cancer. Data were downloaded from TCGA via the UCSC Xena platform, while R package “ESTIMATE” was used to evaluate the immune score, stromal score, and tumor purity score. Subsequently, the specific tumor-infiltrating immune cells (TIICs) and its correlation to BRIP1 expression were assessed via Tumor Immune Estimation Resource (TIMER) database (https://timer.cistrome.org/) [17]. The TIMER, EPIC, MCPCOUNTER, CIBERSORT, CIBERSORT_ABS, XCELL, and QUANTISEQ algorithms were utilized to estimate the immune infiltration of the 21 TIICs. Relationship between BRIP1 expression and immune-related genes was also evaluated at the pan-cancer level. The visualization of the results was implemented with R packages “ggplot2” and “pheatmap.” Besides, TMB, which reflects cancer mutation quantity, has been considered as a leading candidate biomarker for immune checkpoint blockade (ICB) [18]. Meanwhile, MSI, which facilitates mutation and acts as a biomarker of response to immune checkpoint inhibitors (ICPis), plays an important role in improving the possibility of a favorable response to immunotherapy [19]. We thus analyzed the TMB-MSI association with BRIP1 in pan-cancer by Pearson correlation using Sangerbox tools (https://vip.sangerbox.com/home.html), and the results were shown in radar maps.

### 2.6. BRIP1 Expression and Different Therapies

To further validate the relationship between BRIP1 and ICB therapy response, data from the IMvigor210 cohort, which contains 298 metastatic urothelial cancer cases treated by atezolizumab (an antiprogrammed cell death ligand 1, anti-PD-L1 agent), were obtained and analyzed [20]. Patients were divided into two subgroups, one with a low level of BRIP1 and the other with a high level of BRIP1, according to the best cut-off value identified by the “survminer” R package, and immunotherapy response of BRIP1 was then validated. A chi-square test was carried out to assess the proportion differences of responses between subgroups. Furthermore, relationships between BRIP1 and IC50 of numerous antitumor drugs were explored via the Genomics of Drug Sensitivity in Cancer (GDSC) database (https://www.cancerrxgene.org). A Spearman correlation was used to evaluate the drug resistance.

## 3. Results and Discussion

### 3.1. BRIP1 Expression Profile

**Figure 1:**
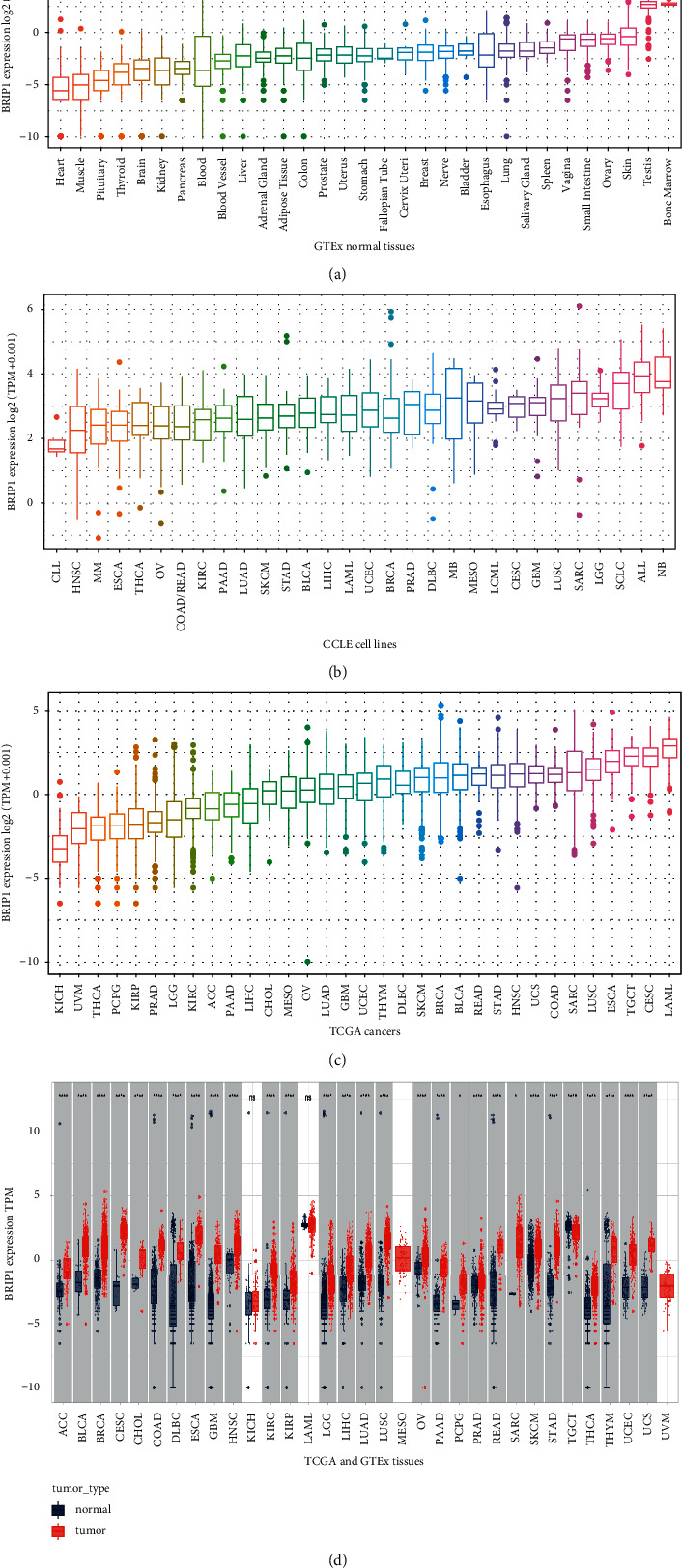
Differential expression level of BRIP1. (a) Expression of BRIP1 in 31 normal tissues from GTEx database. (b) Expression of BRIP1 in 30 cancer cell lines from CCLE database. (c) Expression of BRIP1 in 33 types of cancer from TCGA database. (d) Comparison between tumor and normal tissues of the BRIP1 expression from TCGA and GTEx database. Normalized expression levels of BRIP1 were changed by log_2_(TPM + 0.001). ^*∗*^ represents *P* < 0.05, ^*∗∗*^ represents *P* < 0.01, ^*∗∗∗*^ represents *P* < 0.001, and ^*∗∗∗∗*^ represents *P* < 0.0001. Expression levels of BRIP1 in the first three figures are arranged in ascending order.

**Figure 2:**
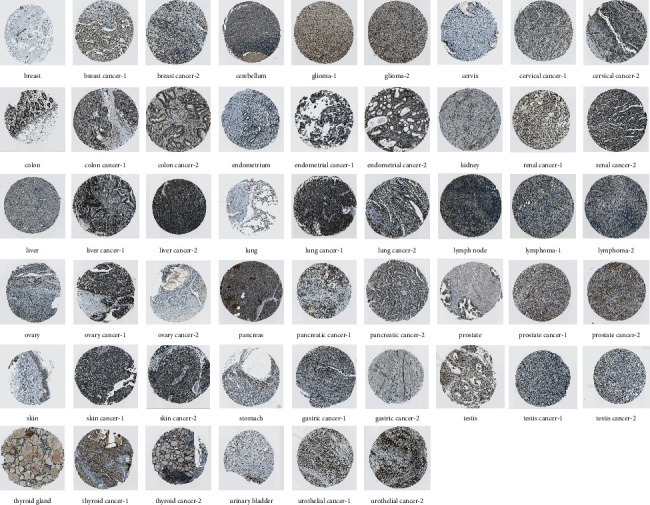
Representative IHC images of BRIP1 in normal and tumor tissues from HPA database.

The expression level of BRIP1 explored via the GTEx transcriptomics dataset indicated it was low in most normal tissues under physiological circumstances, whereas higher in bone marrow than other 30 tissues (Figure 1(a)). Results from CCLE revealed that its expression level was generally increased in various cancer cell lines as the highest expression was in NB, ALL, and SCLC (Figure 1(b)). TCGA data showed a similar expression tendency to that of CCLE, and the highest expression level of BRIP1 was in LAML and genital cancers such as CESC and TGCT (Figure 1(c)). Comparison of the expression level between cancer and normal tissues combing TCGA and GTEx data manifested that BRIP1 was significantly upregulated in 7 digestive tumors (including CHOL, COAD, ESCA, LIHC, PAAD, READ, and STAD) and other 21 tumors (including ACC, BLCA, BRCA, CESC, DLBC, GBM, HNSC, KIRC, KIRP, LGG, LUAD, LUSC, OV, PCPG, PRAD, SARC, SKCM, THCA, THYM, UCEC, and UCS) and downregulated in TGCT (Figure 1(d)), indicating that BRIP1 might play an oncogenic role during carcinogenesis and may function as a potential diagnostic biomarker. Moreover, a noteworthy increase in the expression of BRIP1 was detected in 16 cancers (including BLCA, BRCA, CESC, CHOL, COAD, ESCA, HNSC, KIRC, KIRP, LIHC, LUAD, LUSC, READ, STAD, THCA, and UCEC) between paired tumor tissues and their corresponding normal tissues (Figure S1). When further seeking for the association between BRIP1 expression and different tumor stages, we found that there was a significant difference between stage I, II and stage III, IV in ACC, KIRP, LUAD, and OV (Figure S2). Subsequently, the protein level of BRIP1 was explored in multiple tumor and normal tissues. Representative IHC images showed that BRIP1 was mostly enriched in the nucleoplasm and nuclear membrane and had a low expression level in normal tissues than that of tumor tissues in breast, cerebellum, cervix, colon, endometrium, kidney, liver, lung, lymph node, ovary, pancreas, prostate, skin, stomach, thyroid gland, and urinary bladder, while high in normal testis tissues than tumor tissues (Figure 2).

### 3.2. Prognostic Value of BRIP1 across Cancers

**Figure 3:**
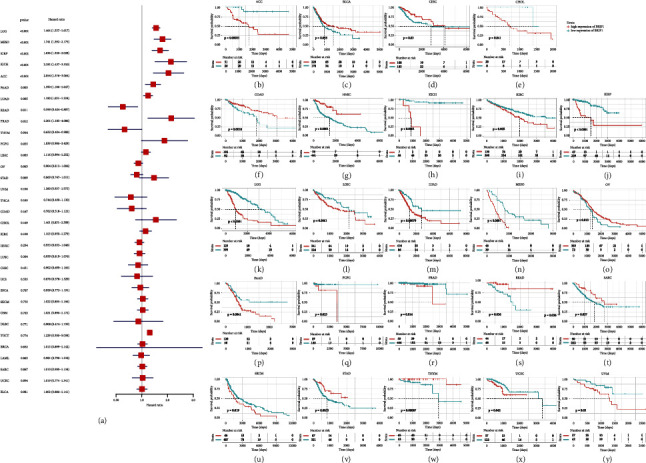
Correlation between BRIP1 expression and overall survival (OS). (a) Forest plot of associations between BRIP1 and OS in 33 cancer types. (b–y) KM analysis results of the relationship between BRIP1 level and OS. The high and low expression level of BRIP1 was divided by the best cut-off value.

Given the aberrant expression of BRIP1 observed in pan-cancer, we wonder its role within prognosis. Therefore, we analyzed the expression of BRIP1 and its association with OS, DSS, DFI, and PFI, respectively. Cox proportional hazards model analysis elucidated BRIP1 expression was correlated with OS in LGG (*P* < 0.001), MESO (*P* < 0.001), KIRP (*P* < 0.001), KICH (*P* < 0.001), ACC (*P* < 0.001), PAAD (*P*=0.003), LUAD (*P*=0.005), READ (*P*=0.011), PRAD (*P*=0.012), and THYM (*P*=0.044). BRIP1 was a high-risk factor in LGG, MESO, KIRP, KICH, ACC, PAAD, LUAD, and PRAD, while it was a low-risk factor in READ and THYM. These results are shown in forestplot in Figure 3(a). KM survival analyses illustrated that upregulated BRIP1 was associated with poor OS in ACC, CHOL, KICH, KIRC, KIRP, LGG, LIHC, LUAD, MESO, PAAD, PCPG, PRAD, SKCM, UCEC, and UVM, while downregulated BRIP1 had shorter survival times in BLCA, CESC, COAD, HNSC, OV, READ, SARC, STAD, and THYM (Figures 3(b)–3(y)).

**Figure 4:**
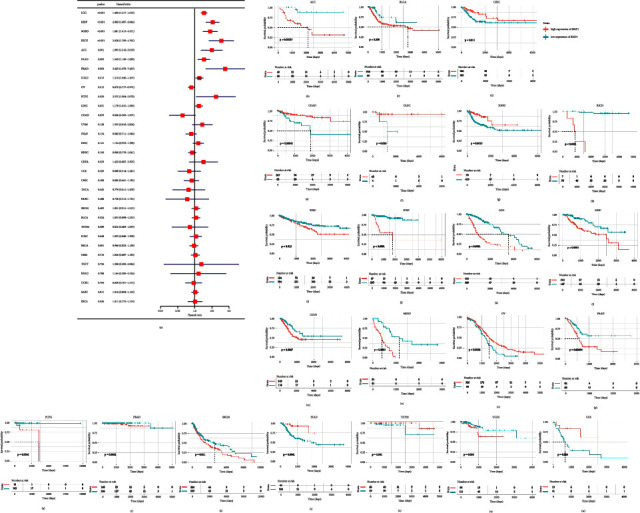
Correlation between BRIP1 expression and disease-specific survival (DSS). (a) Forest plot of associations between BRIP1 and DSS in 33 cancer types. (b–w) KM analysis results of the relationship between BRIP1 level and DSS. The high and low expression level of BRIP1 was divided by the best cut-off value.

As for DSS, it was associated with BRIP1 in LGG (*P* < 0.001), KIRP (*P* < 0.001), MESO (*P* < 0.001), KICH (*P* < 0.001), ACC (*P*=0.001), PAAD (*P*=0.002), PRAD (*P*=0.002), LUAD (*P*=0.013), OV (*P*=0.015), PCPG (*P*=0.029), and LIHC (*P*=0.031), among which BRIP1 was considered as a low-risk factor in OV and a high-risk factor in other cancer types (Figure 4(a)). Besides, worse DSS was found in ACC, BLCA, KICH, KIRC, KIRP, LGG, LIHC, LUAD, MESO, PAAD, PCPG, PRAD, SKCM, and UCEC with the increased expression level of BRIP1, while in CESC, COAD, DLBC, HNSC, OV, STAD, THYM and UCS with the decreased expression level of BRIP1 (Figures 4(b)–4(w)).

**Figure 5:**
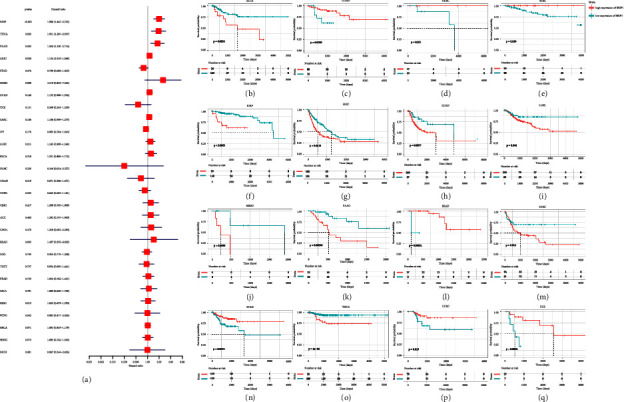
Correlation between BRIP1 expression and disease-free interval (DFI). (a) Forest plot of associations between BRIP1 and DFI in 33 cancer types. (b–q) KM analysis results of the relationship between BRIP1 level and DFI. The high and low expression level of BRIP1 was divided by the best cut-off value.

When considering the relationship between BRIP1 expression and DFI, there was a significant association between them in KIRP (*P* < 0.001), THCA (*P*=0.002), PAAD (*P*=0.003), and LIHC (*P*=0.026). Moreover, BRIP1 was a high-risk factor in all of these four cancers (Figure 5(a)). In addition, poor DFI was perceived in BLCA, KIRP, LIHC, LUAD, LUSC, MESO, PAAD, SARC, and THCA as BRIP1 upregulated in these tumors, while in COAD, DLBC, KIRC, READ, STAD, UCEC, and UCS as BRIP1 downregulated (Figures 5(b)–5(q)).

**Figure 6:**
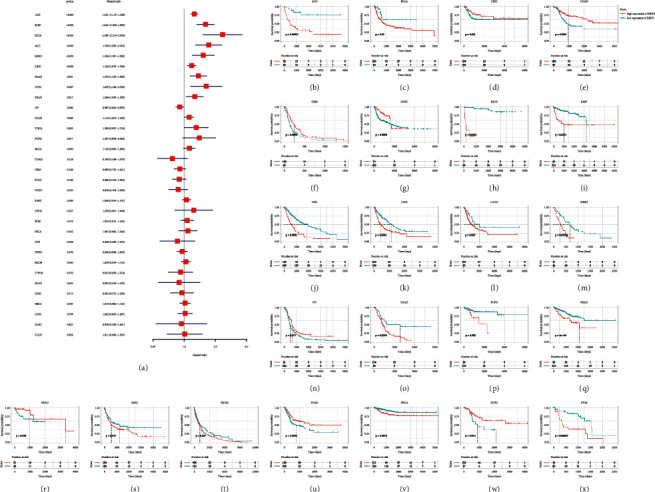
Correlation between BRIP1 expression and progression-free interval (PFI). (a) Forest plot of associations between BRIP1 and PFI in 33 cancer types. (b–x) KM analysis results of the relationship between BRIP1 level and PFI. The high and low expression level of BRIP1 was divided by the best cut-off value.

Regarding PFI, it was correlated with BRIP1 in LGG (*P* < 0.001), KIRP (*P* < 0.001), KICH (*P* < 0.001), ACC (*P* < 0.001), MESO (*P* < 0.001), LIHC (*P* < 0.001), PAAD (*P*=0.001), UVM (*P*=0.007), PRAD (*P*=0.017), OV (*P*=0.040), and LUAD (*P*=0.048), among which BRIP1 was regarded as a low-risk factor in OV but a high-risk factor in others (Figure 6(a)). Additionally, increased expression of BRIP1 was associated with poor PFI in ACC, BLCA, HNSC, KICH, KIRP, LGG, LIHC, LUAD, MESO, PAAD, PCPG, PRAD, SARC, SKCM, THCA, and UVM, while its decreased expression was correlated to poor PFI in CESC, COAD, GBM, OV, READ, STAD, and UCEC (Figures 6(b)–6(x)).

In general, BRIP1 expression level was a vital factor influencing the survival of various cancers and it played an important role in the tumor progression and recurrence.

### 3.3. Correlation between BRIP1 Expression and CNV as well as DNA Methylation in Pan-Cancer

**Figure 7:**
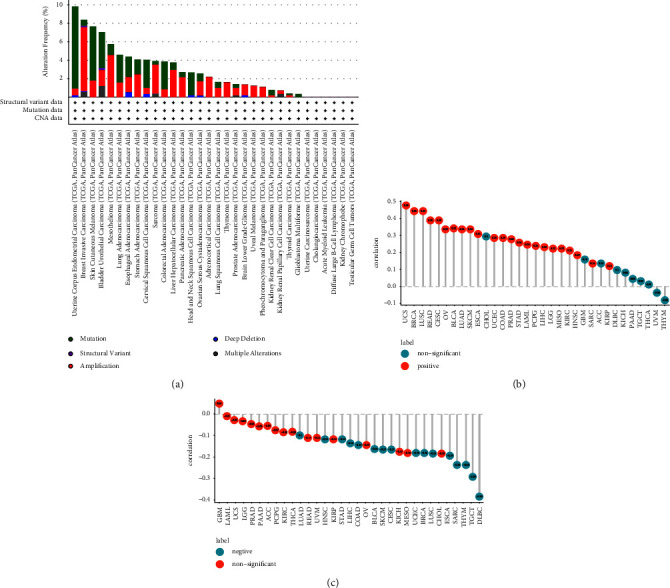
Genetic mutation of BRIP1 and its correlation with CNV and DNA methylation. (a) BRIP1 alteration frequency in pan-cancer. (b) Correlation between BRIP1 expression and CNV. (c) Correlation between BRIP1 expression and methylation.

The previous findings indicated that BRIP1 might play a role in the carcinogeneses and it was widely accepted that the genomic mutation was associated with tumorigenesis. Therefore, a comparative analysis of genomic mutations of BRIP1 in pan-cancer was conducted. Results from the cBioPortal database consisting of 32 cancer types and 10953 tumor samples showed that the amplification of BRIP1 was one of the most vital single factors for alteration. It accounted for 6.92%, 4.6%, and 3.14% in BRCA, MESO, and sarcoma, respectively, as the largest proportion of all mutation types among these tumors (Figure 7(a)). Meanwhile, mutation of BRIP1 became the most important single factor for alteration in UCEC (8.88%), SKCM (5.86%), and BLCA (3.89%). Moreover, there was a significant positive correlation between CNV and BRIP1 expression in 5 digestive tumors (COAD, ESCA, LIHC, STAD, and READ) and other 18 tumors (UCS, BRCA, LUSC, CESC, OV, BLCA, LUAD, SKCM, UCEC, PRAD, LAML, PCPG, LGG, MESO, KIRC, HNSC, SARC, and KIRP), as shown in the lollipop chart (Figure 7(b)), and the correlation in each specific tumor type was summarized in Figure S3. As for promoter methylation, it was significantly negatively associated with the expression level of BRIP1 in 4 digestive tumors (COAD, ESCA, LIHC, and STAD) and other 12 tumors (LUAD, HNSC, BLCA, SKCM, CESC, UCEC, BRCA, LUSC, SARC, THYM, TGCT, and DLBC) (Figures 7(c) and S4).

### 3.4. Interacting Genes of BRIP1 and Its Enrichment and Variation Analysis

**Figure 8:**
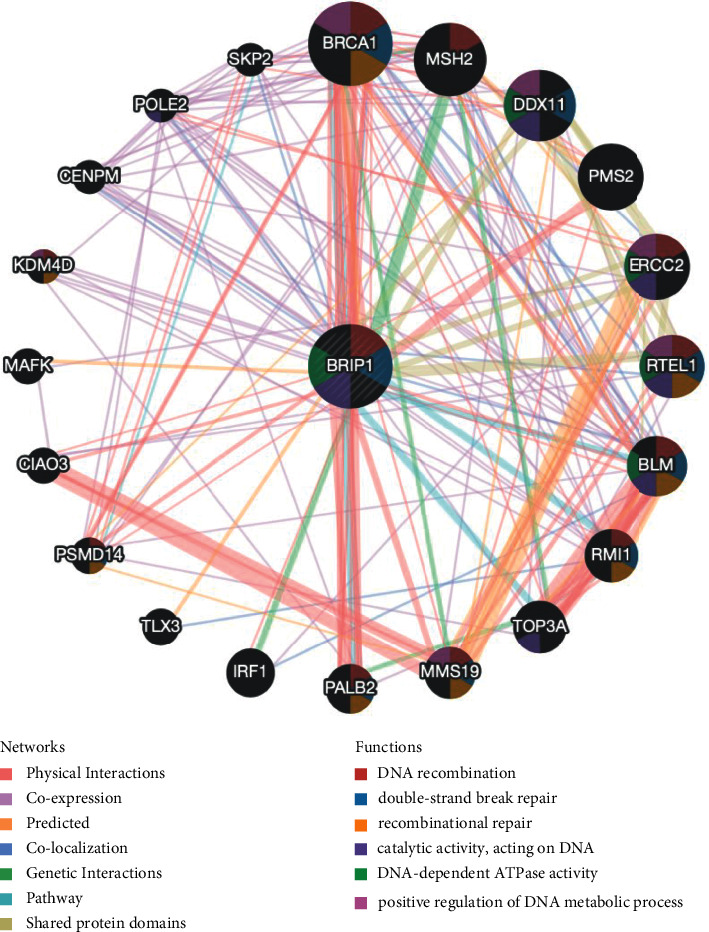
The PPI network and function analysis of BRIP1 from GeneMANIA.

The PPI network for BRIP1 and its coexpressed as well as colocalized genes were constructed by GeneMANIA. The results showed the 20 most frequently altered proteins closely linked to BRIP1, in which BRCA1 had the most prominent correlation with BRIP1 as expected. Besides, the functional analysis indicated that BRIP1 and its similar genes had a significant association with DNA recombination, double-strand break repair, and recombinational repair (Figure 8). To uncover the function of BRIP1, we carried out GSEA in 33 cancer types, and the results suggested that the top 6 signalling pathways correlated with BRIP1 among all cancers based on KEGG were DNA replication, cell cycle, spliceosome, nucleocytoplasmic transport, homologous recombination, and Fanconi anemia pathway. The specific 20 signalling pathways associated with BRIP1 in each type of tumor are summarized in Figure S5.

**Figure 9:**
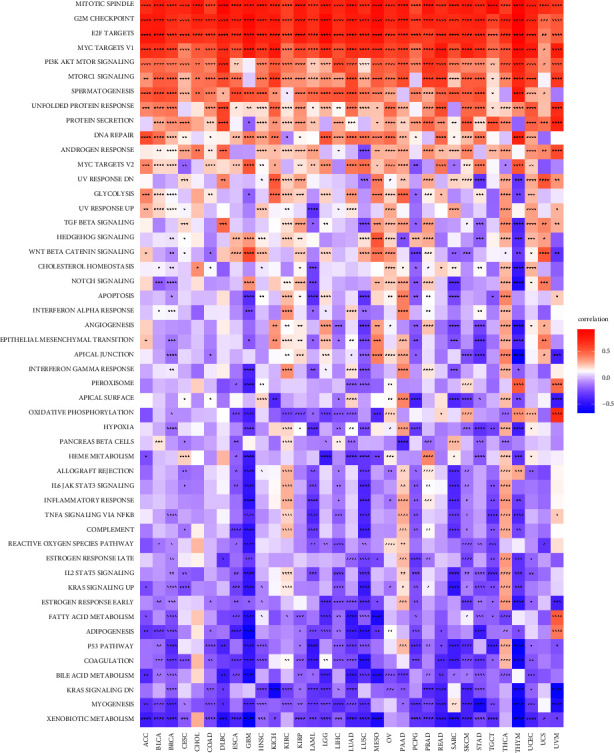
GSVA of BRIP1 in pan-cancer. ^*∗*^ represents *P* < 0.05, ^*∗∗*^ represents *P* < 0.01, ^*∗∗∗*^ represents *P* < 0.001, and ^*∗∗∗∗*^ represents *P* < 0.0001.

As for GSVA, the relationship between BRIP1 and various hallmark pathways in pan-cancer is shown in the heatmap (Figure 9). It was obvious that BRIP1 had the most significantly positive correlation with G2M checkpoint and E2F targets in ACC, BLCA, LGG, LUSC, PCPG, and THYM, with mitotic spindle and G2M checkpoint in BRCA, ESCA, MESO, OV, PAAD, and PRAD, with mitotic spindle in CESC, CHOL, DLBC, SARC, TGCT, and UCS, with G2M checkpoint in COAD, GBM, STAD, and THCA, with mitotic spindle, G2M checkpoint, and E2F targets in HNSC, KIRC, KIRP, LAML, LIHC, and LUAD, with G2M checkpoint and MYC targets V1 in KICH, with MYC targets V1 in READ and UCEC, with mitotic spindle and MYC targets V1 in SKCM, and with MYC targets V1 and protein secretion in UVM. Moreover, the most prominently negative correlation between BRIP1 and xenobiotic metabolism lay in ACC, BLCA, DLBC, READ, SARC, and UCEC, between BRIP1 and xenobiotic metabolism as well as myogenesis lay in BRCA, between BRIP1 and coagulation as well as KRAS signalling upregulation lay in CESC, between BRIP1 and xenobiotic metabolism and myogenesis as well as P53 pathway lay in COAD, between BRIP1 and xenobiotic metabolism and adipogenesis as well as complement lay in ESCA, between BRIP1 and xenobiotic metabolism as well as adipogenesis lay in GBM, between BRIP1 and KRAS signalling downregulation lay in HNSC, KICH, and UVM, between BRIP1 and KRAS signalling downregulation as well as oxidative phosphorylation lay in KIRC, between BRIP1 and xenobiotic metabolism as well as oxidative phosphorylation lay in KIRP, between BRIP1 and coagulation as well as P53 pathway lay in LAML, between BRIP1 and bile acid metabolism as well as heme metabolism lay in LGG, between BRIP1 and myogenesis lay in LIHC and STAD, between BRIP1 and bile acid metabolism as well as fatty acid metabolism lay in LUAD, between BRIP1 and coagulation as well as adipogenesis lay in LUSC, between BRIP1 and xenobiotic metabolism and bile acid metabolism as well as fatty acid metabolism lay in MESO, between BRIP1 and bile acid metabolism lay in OV, between BRIP1 and pancreas beta cells lay in PAAD, between BRIP1 and apical surface lay in PCPG, between BRIP1 and xenobiotic metabolism as well as KRAS signalling downregulation lay in PRAD, between BRIP1 and xenobiotic metabolism, myogenesis as well as KRAS signalling downregulation lay in SKCM, between BRIP1 and P53 pathway lay in TGCT, between BRIP1 and fatty acid metabolism lay in THCA, and between BRIP1 and xenobiotic metabolism, myogenesis, and P53 pathway as well apical junction lay in THYM. In summary, the previous results elucidated the hallmark pathways and potential mechanisms of BRIP1 in pan-cancer. In essence, BRIP1 kept an intimate relationship with HRR, cell cycle, and varied metabolism in different cancers.

### 3.5. BRIP1 Expression and Its Correlation with TME and Immune Infiltration

**Figure 10:**
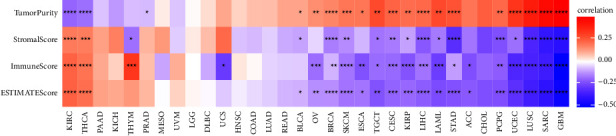
Correlation between BRIP1 expression and TME evaluated by ImmuneScore, StromalScore, and ESTIMATEScore. ^*∗*^ represents *P* < 0.05, ^*∗∗*^ represents *P* < 0.01, ^*∗∗∗*^ represents *P* < 0.001, and ^*∗∗∗∗*^ represents *P* < 0.0001.

**Figure 11:**
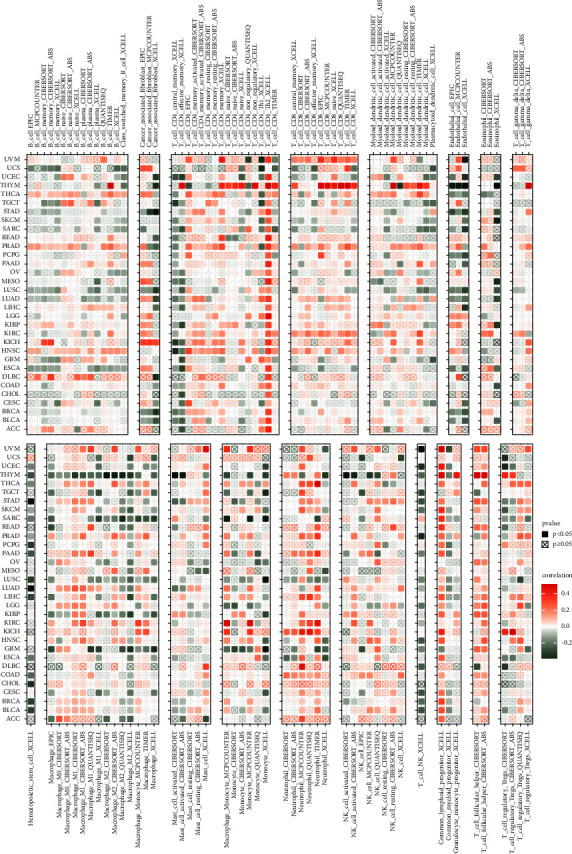
BRIP1 expression and its association with TIICs from TIMER database.

Along with the above coexpressed genes and signalling pathways, TME and immune infiltration also take part in the regulation of tumorigenesis. As part of the complex microenvironment, TIICs have a crucial role in cancer progression and therapeutic responses. Accordingly, we explored the correlation between BRIP1 expression and TME by ESTIMATE and evaluated the coefficient of BRIP1 expression and immune infiltration level via TIMER. The results revealed that the expression of BRIP1 had significant correlations with tumor purity and ESTIMATEScore in 19 cancer types (Figure 10). The top three most significant cancers associated with BRIP1 expression were GBM, SARC, and LUSC based on ImmuneScore, StromalScore, and ESTIMATEScore. The higher the level of BRIP1 as these three tumors expressed, the less stromal and immune cells as these tumors had. On the contrary, the higher level of BRIP1 as these tumors expressed, the higher purity as these tumors had. Results of ESTIMATEScore for all tumor types are listed in Figure S6. In addition, BRIP1 expression and its association with TIICs were significant in most cancer types (Figure 11). Especially in THYM, BRIP1 had a positive correlation with B cells, memory and naive CD4+ T cells, CD8+ T cells, myeloid dendritic cells, neutrophils (by CIBERSORT, CIBERSORT_ABS, and TIMER algorithm), common lymphoid progenitor, granulocyte and monocyte progenitor, and a negative correlation with fibroblast, endothelial cell, eosinophil, macrophage, mast cell, monocyte, neutrophil (by XCELL and MCPCOUNTER algorithm), NK cell, and common myeloid progenitor. As for those digestive tumors, the most significant association between BRIP1 and TIICs was found in STAD. A significantly negative correlation was found between BRIP1 and fibroblast as well as hematopoietic stem cell in STAD.

**Figure 12:**
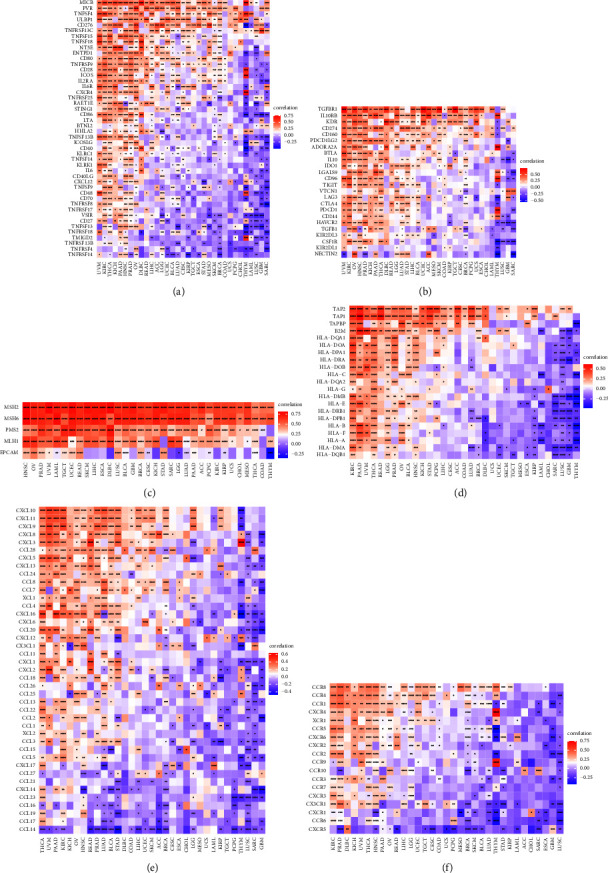
Correlation between BRIP1 and immune-related genes in pan-cancer. (a) Association between expression of BRIP1 and immune-activating genes. (b) Correlation between BRIP1 expression and immunosuppressive genes. (c) BRIP1 expression and its relationship with MMR genes. (d) BRIP1 expression and correlation with genes encoding MHC. (e) Association between BRIP1 expression and chemokine. (f) Correlation between expression of BRIP1 and chemokine receptor proteins. ^*∗*^ represents *P* < 0.05, ^*∗∗*^ represents *P* < 0.01, ^*∗∗∗*^ represents *P* < 0.001, and ^*∗∗∗∗*^ represents *P* < 0.0001.

Moreover, the association between the expression of BRIP1 and immune-related genes including immune-activating genes, immunosuppressive genes, mismatch repair (MMR) genes, and genes encoding the major histocompatibility complex (MHC), chemokine, and chemokine receptor proteins is evaluated (Figure 12). Results indicated that BRIP1 was positively correlated with the majority of immune-activating genes as well as immunosuppressive genes in UVM, KIRC, THCA, KICH, PAAD, HNSC, PRAD, and OV. In HNSC, OV, PRAD, UVM, LAML, TGCT, UCEC, READ, SKCM, LIHC, ESCA, DLBC, LUSC, BLCA, GBM, BRCA, CESC, KICH, STAD, SARC, LGG, PAAD, PCPG, and KIRC, the expression of BRIP1 was positively correlated with most of the MMR genes. As for the correlation between BRIP1 and the majority of genes encoding the chemokine and chemokine receptor proteins, a positive correlation was found in THCA for the former and in KIRC and PRAD for the latter. Besides, there was a positive correlation between BRIP1 and most of the MHC-related genes in KIRC, PAAD, UVM, and THCA, and a negative correlation between BRIP1 and most of the MHC-related genes in THYM, GBM, and LUSC.

### 3.6. Correlation between BRIP1, TMB/MSI, and Immunotherapy Response

**Figure 13:**
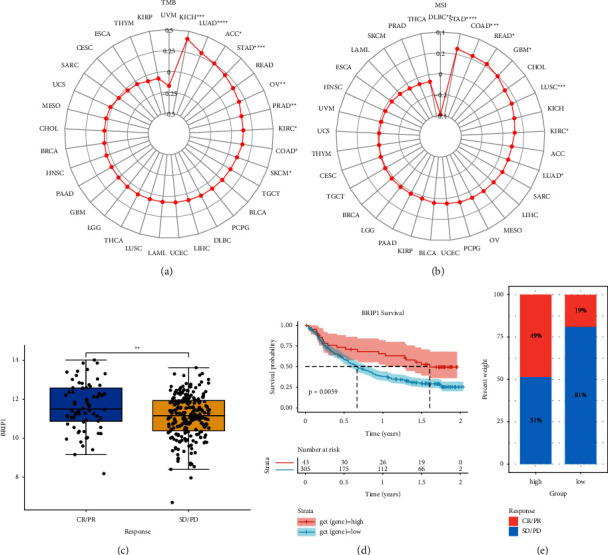
Correlation of BRIP1 expression with TMB/MSI and immunotherapy response. (a) Correlation between BRIP1 and TMB in pan-cancer. (b) Correlation between BRIP1 and MSI in pan-cancer. (c) Association between PD-L1 treatment response and BRIP1 in IMvigor210 cohort. (d) KM curve of the relationship between BRIP1 and survival rate in IMvigor210 cohort. (e) Response rate to PD-L1 therapy in different BRIP1 expression subgroups of IMvigor210 cohort. CR represents complete response, PR represents partial response, PD represents progressive disease, and SD represents stable disease. ^*∗*^ represents *P* < 0.05, ^*∗∗*^ represents *P* < 0.01, ^*∗∗∗*^ represents *P* < 0.001, and ^*∗∗∗∗*^ represents *P* < 0.0001.

To discover the role of BRIP1 in predicting the response to ICPis, we assessed the correlation between BRIP1 expression and the two famous biomarkers, TMB and MSI. BRIP1 was positively associated with TMB in 2 digestive tumors (COAD and STAD) and other 7 tumors including KICH, LUAD, ACC, OV, PRAD, KIRC, and SKCM (Figures 13(a) and S7). As for MSI, it was negatively correlated with BRIP1 in DLBC and positively correlated with BRIP1 in 3 digestive tumors (COAD, READ, and STAD) and other 4 tumors including GBM, LUSC, KIRC, and LUAD (Figures 13(b) and S8). As indicated by previous studies that high TMB/MSI-H increased patients' response to ICPis and was correlated to better immunotherapy outcomes, we therefore, downloaded data of the IMvigor210 cohort to investigate the correlation between BRIP1 and treatment response. Results showed that in this urothelial cancer cohort, patients with a high level of BRIP1 had a better response to the treatment and a more favorable survival rate (Figures 13(c) and 13(d)). Moreover, the anti-PD-L1 response rate was 49% among patients with a high expression level of BRIP1, while there were only 19% of the low-BRIP1 patients responding to the treatment (Figure 13(e)). These results showed the potential of BRIP1 in predicting immunotherapy response and BRIP1 could be a promising candidate biomarker for immunotherapy of various cancers.

### 3.7. BRIP1 and Antitumor Drugs

Other than immunotherapy, the relationships between BRIP1 and IC50 of numerous antitumor drugs are also evaluated (Table S1). Among the 192 antitumor drugs, 141 of them including Olaparib and Niraparib (two PARP inhibitors) were negatively correlated with BRIP1, which indicated a promising response in these treatments. Besides, 7 drugs including Trametinib, SCH772984, ERK_2440, ERK_6604, Selumetinib, Ulixertinib, and VX-11e were positively correlated with BRIP1 which indicated a potential resistance during treatment.

## 4. Discussion

Cancer is a complex polyfactorial disease with high morbidity and mortality, remaining as an unsolved threaten to human health. Thus, research of effective diagnostic biomarkers and therapeutic targets for tumors has always been a heated focus. With the availability of public databases, cancer-related data can be mined to explore novel biomarkers. Through pan-cancer analysis, BRIP1 emerged from a bunch of candidate genes who were applicable for broad-spectrum tumor diagnosis as it significantly upregulated in most tumors. Herein, we conducted a systematic and comprehensive analysis of BRIP1 in pan-cancer. We validated its differential expression in various cancers between tumor and normal tissues at transcriptional and protein levels. Subsequently, we elucidated its role in prognosis, gene function, and regulatory pathways, and we discovered its association with TME, immune infiltration, immune-related genes, and treatment responses.

BRIP1, with a length of more than 180 kb, is located on chromosome 17q23.2 and encodes a protein of 1249 amino acids. Previous studies regarded BRIP1 as a tumor suppressor gene and revealed its diagnostic role in various types of cancer, such as breast cancer, ovarian cancer, cervical cancer, and colon cancer [21–24]. In our comprehensive data mining-based analysis, by analyzing data from the GTEx, CCLE, and TCGA databases, we revealed that BRIP1 expression was higher in 28 types of cancer tissues (including BRCA, CESC, COAD, and OV, in consistence with previous study results) and only lower in TGCT than in normal tissues. Furthermore, our results of differential expression analysis of paired samples and the results of IHC analysis also confirmed the diagnostic role of BRIP1 in pan-cancer. Unfortunately, due to the lack of normal sample data, differential expression analysis could not be conducted in MESO and UVM. Accumulating evidence will be needed for further exploration in these two tumors. Besides, we found a significant differential expression between tumor stage I, II and stage III, IV in ACC, KIRP, LUAD, and OV, suggesting the predicting role of BRIP1 in early diagnosis of these cancers is worth looking forward to. Along with its predicting role in diagnosis, we also performed prognostic analyses in pan-cancer based on data from TCGA. Either from OS or DSS, as well as from DFI and PFI, we found a significant correlation between BRIP1 expression and survival probability in various cancers, among which, BRIP1 was basically a high-risk factor. Whether it be OS, DSS, DFI, or PFI, BRIP1 remained as a high-risk factor in KIRP and PAAD. Although our results from TCGA database did not find a correlation between BRIP1 expression and prognosis of breast cancer patients, a study based specifically on several breast cancer databases exhibited that higher BRIP1 expression was correlated with poor OS, DSS, DFI, and PFI [25]. Another study mining data of LUAD patients from the Genomic Data Commons (GDC) Data Portal indicated that BRIP1 might regulate fibroblast growth factor 22 and affect MAPK as well as Rap 1 signalling pathways in all tumor stages of LUAD, and a high level of BRIP1 showed boundary significance on OS [26], in consistence with our results. Synthesizing the previous results, we believed that the high expression of BRIP1 could hamper cancer patients' survival and it might be an independent prognostic factor for various tumors. Although BRIP1 seemed to be a novel biomarker of vital clinical utility in predicting diagnosis and prognosis in pan-cancer, the distinct effects of the differential expression of BRIP1 on protein function in various cancer types remain largely unknown. Previously, a meta-analysis based on 29400 patients with 116000 controls from 63 studies found BRIP1 was associated with a high risk of ovarian cancer and the HRR pathway might be involved [27, 28]. A cohort of more than 117000 patients elucidated the missense variant of BRIP1 conferred risk for ovarian and breast cancer. Researchers further studied the functional characterization of BRIP1, revealing an impaired interstrand crosslink (ICL) repair of DNA due to the missense variants of BRIP1 [29]. In an Asian esophageal squamous cell carcinoma cohort, researchers found that BRIP1 mutant was an adverse factor for OS and the cohort harboured TP53 signalling pathway alterations altered NOTCH, RTK-RAS, and cell cycle pathway, which might explain the phenomenon [30]. As reported by Singh, via quantitative real-time polymerase chain reaction (qRT-PCR) and Caspase-3 immunostaining, they found that the loss of DNA repair genes expression including BRIP1 in testis correlated with increased apoptosis [31]. To sum up, the distinct effects of the differential expression of BRIP1 in various cancer types may rely on different signalling pathways. Furthermore, *in vivo* and *in vitro* experiments are needed to validate the above findings and elucidate the specific underlying mechanisms of BRIP1 in different types of cancer.

The genomic mutation analysis revealed that the amplification of BRIP1 was one of the most vital single factors for alteration. Interestingly, previous studies reported that amplification of the 17q23 region led to a gain of function in lung cancer, liver cancer, pancreatic cancer, bladder cancer, testis cancer, and ovarian cancer [32]. Since this is the region where BRIP1 locates and with our finding of BRIP1 amplification and its role in pan-cancer, the phenomenon shall be explained to some extent. In addition, PPI analysis revealed that BRIP1 was mainly associated with DNA recombination, double-strand break repair, and recombinational repair. Enrichment analysis uncovered its correlation with homologous recombination, DNA replication, cell cycle, and Fanconi anemia. As indicated by previous studies, BRIP1 took part in HRR and helped in reducing the occurrence and persistence of DSB which was regarded as the last defense against feasibly mutagenic and carcinogenic injury [33]. These might explain the underlying mechanisms of BRIP1 in tumorigenesis and provide a theoretical foundation for the discovery and development of targeted drugs. For example, Hodgson et al. illuminated in their study that ovarian cancer patients with loss-of-function mutations in HRR genes, including BRIP1, would benefit from Olaparib treatment [34]. Our study has evaluated the association between BRIP1 and IC50 of various antitumor drugs via GDSC database and found the same promising response to Olaparib. Except a few of antitumor drugs, there were 141 drugs negatively correlated with BRIP1, which indicated a promising treatment response. Furthermore, clinical trials with different drugs in diverse cancers and research on their targeted signalling pathways are urgently needed to validate effective targeted-therapies.

The cancer-related immune microenvironment was sophisticated and was regarded as the seventh marker feature of cancer [35]. Under normal circumstances, the immune system would recognize and eliminate tumor cells, preventing the invasion and metastasis of tumor cells. However, cancer cells could be subtle and survive the immune supervision by integrating with immune cells, thus restraining the immune system. Under this condition, immunotherapy would restore the normal antitumor immune response. Specifically, ICB therapy showed a remarkable clinical benefit in prolonging patient survival [36]. Immune checkpoints maintained a close correlation with immune cells in TME. Programmed death 1 (PD-1)/PD-L1 was one of the most vital immune checkpoint signalling pathways. Elevated expression of PD-1 and PD-L1 by TIICs was associated with suppression of T cell immune function and poor prognosis in cancer patients [37]. Besides, TMB and MSI were both considered as potential biomarkers for predicting ICB response. In this study, we systematically evaluated the correlation between BRIP1 and TME, TIICs, immune-related genes, as well as TMB-MSI. Results showed that there were close relationships between BRIP1 and various TIICs as well as immune-related genes. Additionally, BRIP1 was intimately correlated with the ESTIMATEScore in 19 cancers and positively associated with TMB in 9 cancers with MSI in 7 cancers, indicating a promising response to ICB therapy in these tumors. Especially in urothelial cancer, patients with a high level of BRIP1 had a better response to anti-PD-L1 treatment and a more favorable survival rate. Our research shed light on BRIP1 as a latent immunotherapy biomarker.

## 5. Conclusions

This study highlights the potential role of BRIP1 in pan-cancer as a predictor for diagnosis, prognosis, and treatment response through in-depth analyses of differential expression, relationships between BRIP1 and different prognostic parameters, gene functions, regulatory pathways, TME, TIICs, immune-related genes, and TMB-MSI as well as anticarcinogen. Furthermore, functional and mechanistic experiments are needed to elucidate the role of BRIP1 in specific cancers.

## Figures and Tables

**Table 1:** Full names and abbreviations of the tumor types from TCGA and CCLE.

Abbreviation	Full name
ACC	Adrenocortical carcinoma
ALL	Acute lymphoblastic leukemia
BLCA	Bladder urothelial carcinoma
BRCA	Breast invasive carcinoma
CESC	Cervical squamous cell carcinoma and endocervical adenocarcinoma
CHOL	Cholangiocarcinoma
CLL	Chronic lymphoblastic leukemia
COAD	Colon adenocarcinoma
DLBC	Lymphoid neoplasm diffuse large B-cell lymphoma
ESCA	Esophageal carcinoma
GBM	Glioblastoma multiforme
HNSC	Head and neck squamous cell carcinoma kidney
KICH	Kidney chromophobe
KIRC	Kidney renal clear cell carcinoma
KIRP	Kidney renal papillary cell carcinoma
LAML	Acute myeloid leukemia
LCML	Chronic myeloid leukemia
LGG	Brain lower grade glioma
LIHC	Liver hepatocellular carcinoma
LUAD	Lung adenocarcinoma
LUSC	Lung squamous cell carcinoma
MB	Medulloblastoma
MESO	Mesothelioma
MM	Multiple myeloma
NB	Neuroblastoma
OV	Ovarian serous cystadenocarcinoma
PAAD	Pancreatic adenocarcinoma
PCPG	Pheochromocytoma and paraganglioma
PRAD	Prostate adenocarcinoma
READ	Rectum adenocarcinoma
SARC	Sarcoma
SCLC	Small cell lung cancer
SKCM	Skin cutaneous melanoma
STAD	Stomach adenocarcinoma
TGCT	Testicular germ cell tumor
THCA	Thyroid carcinoma
THYM	Thymoma
UCEC	Uterine corpus endometrial carcinoma uterine
UCS	Uterine carcinosarcoma
UVM	Uveal melanoma

## Data Availability

The original datasets analyzed in this study can be found in the corresponding websites as indicated in the article. Requests for further access to datasets can be directed to the corresponding author.

## Supplementary Materials

Supplementary Materials Figures S1–S8 are separately uploaded as supplementary materials and figure legends are listed as follows: *Figure S1*. Differential expression of BRIP1 in 16 cancers between paired tumor tissues and their corresponding normal tissues. ^*∗*^ represents *P* < 0.05, ^*∗∗*^ represents *P* < 0.01, ^*∗∗∗*^ represents *P* < 0.001, and ^*∗∗∗∗*^ represents *P* < 0.0001. *Figure S2*. Association between BRIP1 expression and different tumor stages. ^*∗*^ represents *P* < 0.05, ^*∗∗*^ represents *P* < 0.01, and ^*∗∗∗∗*^ represents *P* < 0.0001. *Figure S3*. Positive correlation between BRIP1 expression and CNV in 23 specific tumor types including digestive cancers such as COAD, ESCA, LIHC, READ, and STAD. *Figure S4*. Negative association between BRIP1 expression and methylation in 16 specific tumor types including those digestive cancers such as COAD, ESCA, LIHC, and STAD. *Figure S5*. GSEA of BRIP1 based on KEGG in pan-cancer. (A1–A6) GSEA in ACC, BLCA, BRCA, CESC, CHOL, and COAD, respectively. (B1–B6) GSEA in DLBC, ESCA, GBM, HNSC, KICH, and KIRC, respectively. (C1–C6) GSEA in KIRP, LAML, LGG, LIHC, LUAD, and LUSC, respectively. (D1–D6) GSEA in MESO, OV, PAAD, PCPG, PRAD, and READ, respectively. (E1–E6) GSEA in SARC, SKCM, STAD, TGCT, THCA, and THYM, respectively. (F1–F3) GSEA in UCEC, UCS, and UVM, respectively. *Figure S6*. Association between BRIP1 expression and ESTIMATEScore in 33 tumors. (A1–A6) Association between BRIP1 expression and ESTIMATEScore in ACC, BLCA, BRCA, CESC, CHOL, and COAD, respectively. (B1–B6) Association between BRIP1 expression and ESTIMATEScore in DLBC, ESCA, GBM, HNSC, KICH, and KIRC, respectively. (C1–C6) Association between BRIP1 expression and ESTIMATEScore in KIRP, LAML, LGG, LIHC, LUAD, and LUSC, respectively. (D1–D6) Association between BRIP1 expression and ESTIMATEScore in MESO, OV, PAAD, PCPG, PRAD, and READ, respectively. (E1–E6) Association between BRIP1 expression and ESTIMATEScore in SARC, SKCM, STAD, TGCT, THCA, and THYM, respectively. (F1–F3) Association between BRIP1 expression and ESTIMATEScore in UCEC, UCS, and UVM, respectively. *Figure S7*. Correlation between BRIP1 and TMB in 9 tumors including digestive cancers such as COAD and STAD. *Figure S8*. Association between BRIP1 and MSI in 8 tumors including digestive cancers such as COAD, READ, and STAD. *Table S1*. Relationship between BRIP1 and IC50 in pan-cancer.
