# Supplementary material for: Insights into the Oncogenic, Prognostic, and Immunological Role of BRIP1 in Pan-Cancer: A Comprehensive Data-Mining-Based Study
Source: J Oncol. 2023 Apr 28;2023:4104639. doi: 10.1155/2023/4104639 (PMC10162871; doi:10.1155/2023/4104639)
Supplement: Supplementary Materials — Figures S1–S8 are separately uploaded as supplementary materials and figure legends are listed as follows: Figure S1. Differential expression of BRIP1 in 16 cancers between paired tumor tissues and their corresponding normal tissues. ∗ represents P < 0.05, ∗∗ represents P < 0.01, ∗∗∗ represents P < 0.001, and ∗∗∗∗ represents P < 0.0001. Figure S2. Association between BRIP1 expression and different tumor stages. ∗ represents P < 0.05, ∗∗ represents P < 0.01, and ∗∗∗∗ represents P < 0.0001. Figure S3. Positive correlation between BRIP1 expression and CNV in 23 specific tumor types including digestive cancers such as COAD, ESCA, LIHC, READ, and STAD. Figure S4. Negative association between BRIP1 expression and methylation in 16 specific tumor types including those digestive cancers such as COAD, ESCA, LIHC, and STAD. Figure S5. GSEA of BRIP1 based on KEGG in pan-cancer. (A1–A6) GSEA in ACC, BLCA, BRCA, CESC, CHOL, and COAD, respectively. (B1–B6) GSEA in DLBC, ESCA, GBM, HNSC, KICH, and KIRC, respectively. (C1–C6) GSEA in KIRP, LAML, LGG, LIHC, LUAD, and LUSC, respectively. (D1–D6) GSEA in MESO, OV, PAAD, PCPG, PRAD, and READ, respectively. (E1–E6) GSEA in SARC, SKCM, STAD, TGCT, THCA, and THYM, respectively. (F1–F3) GSEA in UCEC, UCS, and UVM, respectively. Figure S6. Association between BRIP1 expression and ESTIMATEScore in 33 tumors. (A1–A6) Association between BRIP1 expression and ESTIMATEScore in ACC, BLCA, BRCA, CESC, CHOL, and COAD, respectively. (B1–B6) Association between BRIP1 expression and ESTIMATEScore in DLBC, ESCA, GBM, HNSC, KICH, and KIRC, respectively. (C1–C6) Association between BRIP1 expression and ESTIMATEScore in KIRP, LAML, LGG, LIHC, LUAD, and LUSC, respectively. (D1–D6) Association between BRIP1 expression and ESTIMATEScore in MESO, OV, PAAD, PCPG, PRAD, and READ, respectively. (E1–E6) Association between BRIP1 expression and ESTIMATEScore in SARC, SKCM, STAD, TGCT, THCA, and THYM, respectively. (F1–F3) Association between BRIP1 expression and E [file 4104639.f1.zip › Table S1 (1).docx]

Table S1. Relationship between BRIP1 and IC50 in pan-cancer

| anti-tumor drugs | correlation between BRIP1 and IC50 | p-value |
| --- | --- | --- |
| Camptothecin | -0.133399169 | 0.00014848 |
| Vinblastine | -0.13827468 | 0.00014997 |
| Cisplatin | -0.144425262 | 6.09x10^-5^ |
| Cytarabine | -0.102086747 | 0.00516531 |
| Docetaxel | -0.095398165 | 0.00030314 |
| Navitoclax | -0.179736624 | 7.63x10^-7^ |
| Vorinostat | -0.182971062 | 4.15 x10^-7^ |
| Nilotinib | -0.131821931 | 0.00028392 |
| Olaparib | -0.156399708 | 1.52 x10^-5^ |
| AZD7762 | -0.180066985 | 5.83 x10^-7^ |
| Afatinib | -0.117720026 | 0.00084349 |
| Staurosporine | -0.100201893 | 0.00541641 |
| Wee1 Inhibitor | -0.157627106 | 1.41 x10^-5^ |
| Mirin | -0.135976461 | 0.00023825 |
| PD173074 | -0.146738945 | 5.18 x10^-5^ |
| Alisertib | -0.18043998 | 9.73 x10^-7^ |
| MK-2206 | -0.101230438 | 0.00501308 |
| Paclitaxel | -0.115161355 | 0.00153745 |
| Crizotinib | -0.098518276 | 0.00760265 |
| Sorafenib | -0.112198729 | 0.00203205 |
| BI-2536 | -0.335397161 | 0.02790211 |
| Irinotecan | -0.1462199 | 3.38 x10^-5^ |
| Oxaliplatin | -0.087940803 | 0.00058838 |
| GSK1904529A | -0.13142539 | 0.0001969 |
| PRIMA-1MET | -0.154281947 | 2.98 x10^-5^ |
| Erlotinib | -0.085208142 | 0.01968423 |
| Niraparib | -0.159765539 | 1.52 x10^-5^ |
| MK-1775 | -0.175015704 | 1.10 x10^-6^ |
| Dinaciclib | -0.111955146 | 0.00253796 |
| Gemcitabine | -0.117141879 | 0.0012809 |
| Bortezomib | -0.086816963 | 0.01717809 |
| Tamoxifen | -0.113107337 | 0.00187985 |
| Fulvestrant | -0.119143432 | 4.09 x10^-6^ |
| EPZ004777 | -0.145200309 | 3.84 x10^-5^ |
| YK-4-279 | -0.149481419 | 4.00 x10^-5^ |
| Daporinad | -0.229622089 | 6.13 x10^-6^ |
| BMS-345541 | -0.138564046 | 0.00018027 |
| AZ960 | -0.155831256 | 2.57 x10^-5^ |
| Talazoparib | -0.140815068 | 0.00011513 |
| XAV939 | -0.146536785 | 7.40 x10^-5^ |
| Temozolomide | -0.175972287 | 1.30 x10^-6^ |
| AZD5438 | -0.134792538 | 0.00027024 |
| IAP_5620 | -0.160702033 | 1.41 x10^-5^ |
| AZD1208 | -0.182955085 | 7.06 x10^-7^ |
| AZD1332 | -0.077819167 | 0.03605058 |
| Ruxolitinib | -0.155563995 | 2.55 x10^-5^ |
| Linsitinib | -0.111327526 | 0.00161213 |
| Epirubicin | -0.113860648 | 0.00180193 |
| Cyclophosphamide | -0.15426813 | 2.26 x10^-5^ |
| Pevonedistat | -0.084417871 | 0.0210266 |
| Uprosertib | -0.097172716 | 0.00018667 |
| LCL161 | -0.154422582 | 2.97 x10^-5^ |
| Alpelisib | -0.139995073 | 7.02 x10^-5^ |
| Taselisib | -0.084692996 | 0.01650549 |
| EPZ5676 | -0.154860005 | 1.11 x10^-5^ |
| IWP-2 | -0.161512543 | 1.24 x10^-5^ |
| Leflunomide | -0.166467018 | 6.51 x10^-6^ |
| Entinostat | -0.185874936 | 4.09 x10^-7^ |
| LGK974 | -0.081276221 | 0.02150218 |
| VE-822 | -0.196654576 | 9.20 x10^-8^ |
| CZC24832 | -0.181997575 | 7.95 x10^-7^ |
| AZD5582 | -0.123269899 | 0.00096446 |
| GSK2606414 | -0.126232529 | 0.00069255 |
| PFI3 | -0.18271427 | 7.18 x10^-7^ |
| PCI-34051 | -0.16830407 | 5.13 x10^-6^ |
| Wnt-C59 | -0.104976906 | 0.00463315 |
| I-BET-762 | -0.139926692 | 0.00015699 |
| RVX-208 | -0.15801845 | 1.92 x10^-5^ |
| OTX015 | -0.128599933 | 0.0005137 |
| GSK343 | -0.155203746 | 2.70 x10^-5^ |
| ML323 | -0.09289551 | 0.01227577 |
| Entospletinib | -0.088303725 | 0.01732014 |
| PRT062607 | -0.130699561 | 0.00041445 |
| AGI-6780 | -0.129390293 | 0.000474 |
| Picolinici-acid | -0.142570376 | 0.00011594 |
| AZD5153 | -0.115029545 | 0.00192073 |
| CDK9_5576 | -0.105679823 | 0.00439139 |
| CDK9_5038 | -0.120657788 | 0.00113351 |
| Eg5_9814 | -0.175270485 | 2.16 x10^-6^ |
| IRAK4_4710 | -0.097406041 | 0.0086326 |
| JAK1_8709 | -0.11847667 | 0.0013836 |
| AZD5991 | -0.213552139 | 6.82 x10^-9^ |
| PAK_5339 | -0.142189225 | 0.00012229 |
| ULK1_4989 | -0.163962849 | 9.24 x10^-6^ |
| IGF1R_3801 | -0.142356498 | 0.00012003 |
| JAK_8517 | -0.161021653 | 1.30 x10^-5^ |
| AZD4547 | -0.096696065 | 0.00792525 |
| Ibrutinib | -0.127286292 | 0.00060756 |
| Zoledronate | -0.201267999 | 4.51 x10^-8^ |
| Carmustine | -0.165290871 | 7.58 x10^-6^ |
| Topotecan | -0.163220189 | 9.87 x10^-6^ |
| Teniposide | -0.191402826 | 2.03 x10^-7^ |
| Mitoxantrone | -0.184565466 | 5.51 x10^-7^ |
| Dactinomycin | -0.062550555 | 0.01672008 |
| Fludarabine | -0.160846101 | 1.33 x10^-5^ |
| Nelarabine | -0.196108066 | 1.02 x10^-7^ |
| Vincristine | -0.181356574 | 9.66 x10^-7^ |
| Podophyllotoxin bromide | -0.152200727 | 3.82 x10^-5^ |
| Gallibiscoquinazole | -0.134768109 | 0.00027094 |
| Elephantin | -0.143462027 | 0.00010492 |
| Sinularin | -0.135097637 | 0.00026163 |
| Sabutoclax | -0.183353067 | 6.79 x10^-7^ |
| LY2109761 | -0.192825021 | 1.64 x10^-7^ |
| MN-64 | -0.135802948 | 0.00024271 |
| KRAS (G12C) Inhibitor-12 | -0.181684386 | 8.31 x10^-7^ |
| MG-132 | -0.077067008 | 0.03261135 |
| BDP-00009066 | -0.086484668 | 0.01791431 |
| Buparlisib | -0.095243141 | 0.00910253 |
| Venetoclax | -0.201121441 | 2.60 x10^-8^ |
| ABT737 | -0.216013092 | 2.39 x10^-9^ |
| AGI-5198 | -0.138006069 | 0.0001514 |
| AZD3759 | -0.095175107 | 0.0087869 |
| AZD5363 | -0.082105396 | 0.02463419 |
| AZD6738 | -0.159331101 | 1.18 x10^-5^ |
| Osimertinib | -0.091100548 | 0.0126223 |
| Cediranib | -0.085914196 | 0.01868731 |
| Ipatasertib | -0.088024903 | 0.01596525 |
| GDC0810 | -0.132182204 | 0.00028626 |
| GSK2578215A | -0.142178305 | 9.44 x10^-5^ |
| I-BRD9 | -0.146021776 | 6.04 x10^-5^ |
| Telomerase Inhibitor IX | -0.09440666 | 0.00973312 |
| MIRA-1 | -0.183836294 | 4.13 x10^-7^ |
| NVP-ADW742 | -0.123178455 | 0.00072916 |
| P22077 | -0.137403711 | 0.0001619 |
| Savolitinib | -0.117360928 | 0.00130207 |
| UMI-77 | -0.173679244 | 1.74 x10^-6^ |
| WIKI4 | -0.127126958 | 0.00049176 |
| MIM1 | -0.160832027 | 9.73 x10^-6^ |
| WEHI-539 | -0.199337584 | 3.76 x10^-8^ |
| BPD-00008900 | -0.131894733 | 0.00029521 |
| Foretinib | -0.099354192 | 0.00657456 |
| BIBR-1532 | -0.136024791 | 0.00019427 |
| Pyridostatin | -0.169344407 | 3.25 x10^-6^ |
| AMG-319 | -0.17219991 | 2.31 x10^-6^ |
| MK-8776 | -0.175417209 | 1.43 x10^-6^ |
| Vinorelbine | -0.146392606 | 5.99 x10^-5^ |
| LJI308 | -0.125655321 | 0.00059702 |
| AZ6102 | -0.074788131 | 0.04155 |
| VE821 | -0.161699821 | 9.72 x10^-6^ |
| AT13148 | -0.122852646 | 0.00079786 |
| JQ1 | -0.374537465 | 0.0094943 |
| Gefitinib | -0.061527998 | 0.09243861 |
| Axitinib | 0.046122449 | 0.75299702 |
| SB216763 | 0.046850288 | 0.65563896 |
| KU-55933 | 0.046026921 | 0.75607215 |
| PLX-4720 | -0.019754035 | 0.57834308 |
| NU7441 | -0.18640148 | 0.20965213 |
| Doramapimod | -0.105519581 | 0.48522395 |
| Nutlin-3a (-) | -0.049746624 | 0.16816457 |
| ZM447439 | -0.049144311 | 0.74287963 |
| RO-3306 | -0.062961355 | 0.67073976 |
| Palbociclib | -0.064606516 | 0.0739306 |
| Dactolisib | 0.002571614 | 0.943985 |
| Pictilisib | -0.03114727 | 0.39025118 |
| AZD8055 | 0.074214437 | 0.47957 |
| PD0325901 | 0.066535627 | 0.05980441 |
| Obatoclax Mesylate | -0.051482895 | 0.16583737 |
| 5-Fluorouracil | -0.036409564 | 0.30308621 |
| Dasatinib | -0.029499794 | 0.41766341 |
| Rapamycin | -0.05924034 | 0.10687854 |
| BMS-536924 | -0.036253857 | 0.32175431 |
| Tozasertib | -0.137646548 | 0.35084236 |
| PF-4708671 | 0.015740339 | 0.91543622 |
| GSK269962A | -0.015306122 | 0.9177609 |
| SB505124 | 0.173913043 | 0.25322656 |
| Dabrafenib | -0.043885045 | 0.22904535 |
| AZD2014 | -0.010162008 | 0.78458854 |
| Sapitinib | -0.062285136 | 0.07829855 |
| Lapatinib | -0.061988734 | 0.08894967 |
| Luminespib | -0.061509522 | 0.08189977 |
| OSI-027 | 0.001607054 | 0.97934661 |
| WZ4003 | -0.048238105 | 0.19419719 |
| Ribociclib | -0.041134752 | 0.78606312 |
| TAF1_5496 | -0.045757759 | 0.21847847 |
| VSP34_8731 | -0.072002879 | 0.05247115 |
| Acetalax | -0.004890972 | 0.89533395 |
| Dihydrorotenone | -0.002664013 | 0.94295405 |
| OF-1 | -0.050758047 | 0.17188607 |
| Afuresertib | -0.070728298 | 0.05316519 |
| AZD8186 | -0.012037783 | 0.74222364 |
| GNE-317 | 0.016468905 | 0.65577287 |
| Sepantronium bromide | -0.063704419 | 0.08145387 |
| GSK591 | -0.061481613 | 0.09422908 |
| AZD6482 | -0.032030527 | 0.8307561 |
| BMS-754807 | 0.171811135 | 0.26477572 |
| Trametinib | 0.178174924 | 6.83 x10^-7^ |
| SCH772984 | 0.131750221 | 0.00018628 |
| ERK_2440 | 0.078944385 | 0.0334398 |
| ERK_6604 | 0.122470393 | 0.00094416 |
| Selumetinib | 0.135250703 | 0.00025741 |
| Ulixertinib | 0.055874038 | 0.0307523 |
| VX-11e | 0.073489777 | 0.04522863 |
